# State of the art in esophagectomy: robotic assistance in the abdominal phase

**DOI:** 10.1007/s13304-020-00937-w

**Published:** 2020-12-31

**Authors:** Eline M. de Groot, Lucas Goense, Jelle P. Ruurda, Richard van Hillegersberg

**Affiliations:** Department of Surgery, University Medical Center Utrecht, POBOX 85500, 3508 GA Utrecht, The Netherlands

**Keywords:** Robotics, Esophagectomy, Minimally invasive esophagectomy, RAMIE, Da vinci

## Abstract

Over the years, robot-assisted esophagectomy gained popularity. The current literature focused mainly on robotic assistance in the thoracic phase, whereas the implementation of robotic assistance in the abdominal phase is lagging behind. Advantages of adding a robotic system to the abdominal phase include robotic stapling and the increased surgeon’s independency. In terms of short-term outcomes and lymphadenectomy, robotic assistance is at least equal to laparoscopy. Yet high quality evidence to conclude on this topic remains scarce. This review focuses on the evidence of robotic assistance in the abdominal phase of esophagectomy.

## Introduction

Esophagectomy with radical lymphadenectomy, generally combined with neoadjuvant therapy, is the main component of treatment for localized esophageal cancer [1,2]. Esophagectomy is a complex and invasive surgical procedure associated with a relatively high morbidity and mortality rate. Hence, during the last decade, there has been a vast development in novel minimally invasive techniques to enhance recovery and decrease postoperative complications [3].

Various approaches have been investigated (including; hybrid, fully minimally invasive and robot-assisted esophagectomy), each with its specific technical advantages and difficulties. In general, minimally invasive surgery results in faster recovery and lower morbidity, especially reduction of pulmonary complications [4–6]. However, conventional minimally invasive procedures can be technically demanding. The inability to articulate and the rigidity of the thoracoscopic instruments technically hinder proper mediastinal lymphadenectomy. As such, robot-assisted esophagectomy has gained popularity during the last decade as it offers a magnified three-dimensional vision, motion scaling, and articulating instruments, allowing precise dissection of the peri-esophageal tissue along all the vital structures. Moreover, its superiority over open esophageal surgery has been demonstrated in a randomized trial [6].

So far, most studies focused on robotic assistance in the thoracic phase of esophagectomy because of the advantages during mediastinal dissection. However, the potential advantages of robotic assistance in the abdominal phase of the procedure have rarely been reported. This is probably due to the technical limitations of the first robotic systems which were less suitable for the abdominal phase [7]. The current robotic systems are better equipped for maneuvers with large amplitude without collisions of the robotic arms allowing implementation in the abdominal phase.

This article reviews the advances and technical development in robot-assisted esophageal surgery, with an emphasis on the abdominal phase.

## The operation

### Robotic 4-armed system

In 2003, esophageal resection was performed with robotic assistance for the first time [8]. The robotic assistance was limited to the thoracic phase as this was considered to be the most difficult part to be performed thoracoscopically. Furthermore, the first generation of robotic systems was not constructed to navigate in the multiquadrant abdominal area. The more recent developed robotic system, the da Vinci Xi, does allow for multiquadrant surgery since it is equipped with instruments with more freedom of movement and designed to prevent the robotic arms from colliding. In addition, the robotic system is equipped with a fourth arm.

**Fig. 1:**
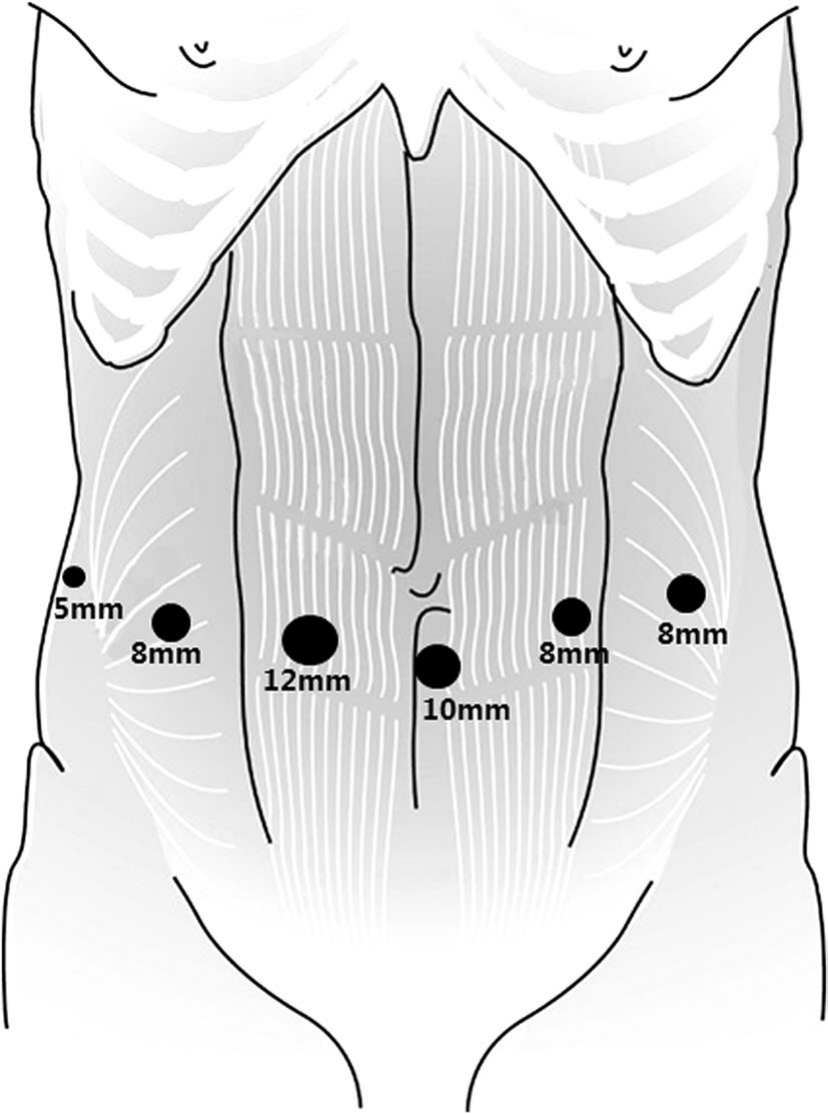
Robotic port positioning of the abdominal phase during esophagectomy. The 10 mm port is used for the table assistant, the 5 mm port for the liver retractor and the 12 mm port for the camera. The other 3 ports are for robotic instruments

The abdominal phase performed with robotic assistance follows the same surgical steps when performed with laparoscopic assistance. The patient is placed in supine position after which 4 robotic ports are inserted (3 × 8 mm and 1 × 12 mm) (Fig. 1). In our center, we make 2 extra entrances; one 5 mm for the liver retractor and one 10 mm for the assistant port.

The lesser omentum is dissected with the Cautery Hook to expose and dissect the crus. Hereafter, the greater curvature and vasa brevia are transected using both Vessel Sealer and the Cautery Hook. The left gastric vein is transected with the vessel sealer and the left gastric artery is clipped with a hem-o-lock before it is divided. The lymph node dissection over the celiac trunk, hepatic, and splenic arteries concludes the abdominal dissection phase.

The creation of the gastric conduit requires optimal surgical proficiency as the fundus of the stomach is sensitive for damage due to grabbing or excessive touching [9]. A disadvantage of robotic assistance, in particular during this part of the surgery, may be the absence of tactile feedback. However, this is solved by lifting and retracting tissues mainly bluntly. Furthermore, robotic surgeons compensate by visual feedback of the tissue behavior upon touch.

**Fig. 2:**
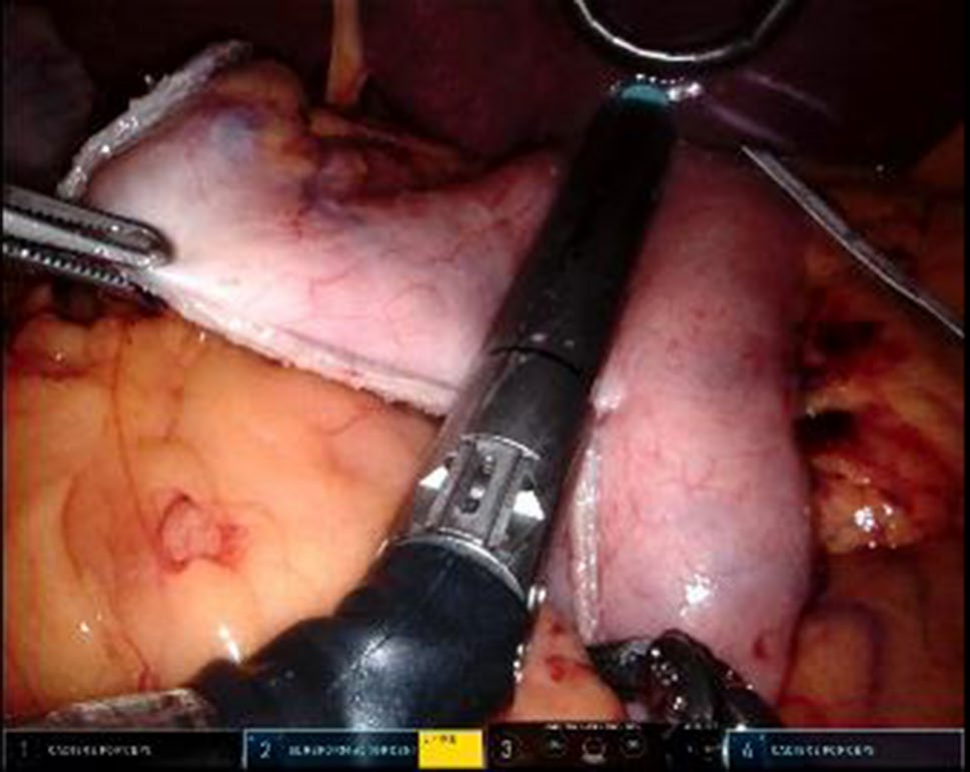
Creation of the gastric conduit during robot-assisted esophagectomy using a 4-armed robotic system and an endowristed stapler device SureForm 60 (Intuitive Surgical Inc, Sunnyvale, CA)

In general, the gastric conduit is created by the use of an endowristed stapler device. This step is technically difficult with laparoscopy because challenging angles have to be made with the stapler. Robotic stapling overcomes this challenge. Robotic surgical tools and stapler devices allow endowristed movements leading to improved surgical dexterity as compared to laparoscopy. The stapler is fully wristed and can articulate up to 100 degrees left and right and more than 50 degrees up and down. Moreover, the stapler provides objective feedback and automatic tissue compression regarding clamp completion [10]. During stapling, the surgeon is able to control the camera, provide traction, and countertraction on the stomach and fire the stapler independently (Fig. 2). These advantages facilitate creation of the gastric conduit and avoid twist of the vertical stapler line by the exact positioning of the robotic staplers.

The surgeon’s independency might be the greatest advantage of a robotic system over laparoscopy in the abdominal phase. During conventional laparoscopy, the surgeon is reliant on an assistant surgeon for both camera view and surgical assistance for the dissection. In particular hospitals where skilled assistants are scarce will benefit from this independency. Taking over these functions by robotic assistance does not fully eliminate table assistance since a robot-specialized scrub nurse remains a key component to successful esophagectomy as inserting and cutting sutures, suctioning, delivery of lymph node stations, and replacing robotic cassettes are still necessary.

The benefits of the 4-armed robotic system for the thoracic phase have been published previously [11]. For the abdominal phase, the 4th arm is used for several purposes. It supports in counter traction by stretching tissue which allows the surgeon to dissect without the aid of an assistant surgeon. In addition, it could create a better exposure by grasping tissue away from the surgical field or it can be used for elevating the left liver lobe [12]. Given these multiple purposes of the 4^th^ arm, it ultimately maximizes the surgeon’s autonomy and independency by taking over the aforementioned tasks which would otherwise be delegated to an assistant surgeon when operating without 4 arms.

Another important advantage of the robotic system is the dexterity in the lymphadenectomy.

## Oncological outcomes

### Lymphadenectomy

Several studies reported on the relevance of an adequate lymph node yield (LNY) during esophagectomy since a higher yield has been associated with increased survival [13,14]. Also on this topic most studies focused on thoracic LNY and only few studies report on abdominal LNY [15].

**Fig. 3:**
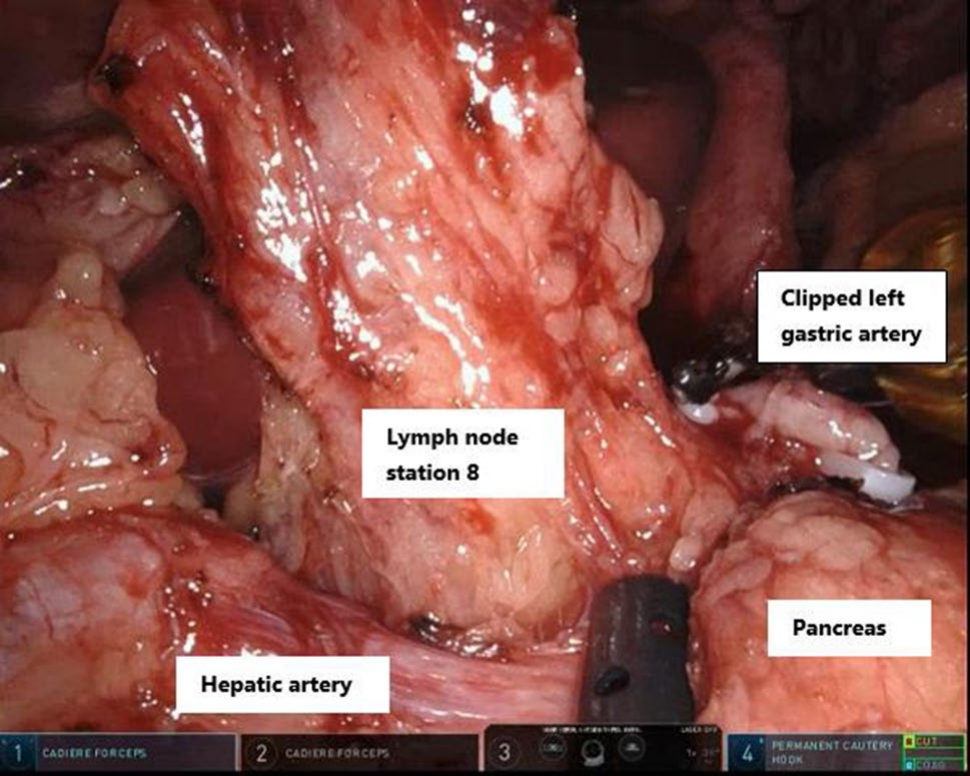
Robot-assisted abdominal lymph node dissection during esophagectomy using a vessel sealer (Intuitive Surgical Inc, Sunnyvale, CA) in robotic arm 1 for lifting station 8 and a Cadiere or Cautery Hook (Intuitive Surgical Inc, Sunnyvale, CA) in arm 2 which could be exchanged during the dissection

In general, lymph nodes are positioned around delicate structures such as the gastroduodenal ligament, gastric artery, hepatic artery, and splenic artery (Fig. 3). Robotic assistance can facilitate proper lymphadenectomy during this part of the procedure due to its technical advantages. A better visualization with a 3-dimensional view, articulating instruments and tremor reduction technology aids in the dissection along these vital structures.

A high-quality lymphadenectomy is expressed in amount of retrieved lymph nodes plus the extent and completeness of the dissection. Regarding the quantity, one might question if the limit of abdominal LNY is already reached by laparoscopy and therefore might not need to improve any further.

Few studies have reported on the quantity of abdominal LNY, comparing robotic assistance to laparoscopy [16–19]. The results are shown in Table 1. Out of the 4 propensity score matched studies, 1 study with 52 patients in both groups, found a significantly higher abdominal LNY with a robot-assisted approach (mean 9.7 ± 6.4 vs. 7.3 ± 5.1, *p* = 0.042) [16]. The other 3 studies did not show any differences in LNY.

**Table 1:** Study characteristics and abdominal lymph node yield and duration of abdominal phase in 5 propensity score matched studies

Author	Procedure	Group 1	Group 2	Patients^#^	Abdominal LNY			Duration of abdominal phase		
				*n* =	Robotic	Laparoscopic	*P* value	Robotic	Laparoscopic	*p* value
Deng et al. [16]	McKeown	Fully robotic	Thoracoscopy–laparoscopy	52 vs. 52	9.7 ± 6.4	7.3 ± 5.1	0.042	94.5 ± 21.6	87.5 ± 20.9	0.096
Kwon et al. [17]	Both	Fully robotic	Thorax: robot-assisted Abdomen: laparoscopy	49 vs. 49	12.4 ± 9.0	12.3 ± 8.9	0.992	NR	NR	NR
Zhang et al. [18]	Ivor Lewis	Fully robotic	Thoracoscopy–laparoscopy	66 vs. 66	8.9 ± 6.7	7.3 ± 5.9	0.198	NR	NR	NR
Yang et al. [19]	McKeown	Fully robotic	Thoracoscopy–laparoscopy	271 vs. 271	7.9 ± 4.8	6.8 ± 3.6	0.237	NR	NR	NR
Tagkalos et al. [36]	Ivor Lewis	Fully robotic	Thoracoscopy–laparoscopy	40 vs. 40	NR	NR	NR	151 (66–335)*	125 (80–250)*	0.001

All outcomes are reported as mean with standard deviation except for *, which is a median with range

^#^Number of patients in matched cohorts

*NR* not reported, *LNY* lymph node yield

However, comparing LNY, especially between studies, is challenging. The determination of LNY is dependent on several factors as delivery and presentation of the lymph nodes to the pathologist. For example, significantly more lymph nodes could be counted when delivered in separate packages instead of en bloc resections [20].

In conclusion, the current evidence showed that robotic assistance contributes to at least an equal harvest of abdominal lymph nodes compared to laparoscopy.

The lymph node dissection could be aided by fluorescence techniques, yet this technology is still in its infancy. Injection of indocyanine green (ICG) in peritumoral tissue has shown to identify lymph nodes structures with a simple switch of camera mode. The camera of robotic systems is commonly equipped with a module for ICG imaging, whereas this is not the case in laparoscopic cameras. Since this technique is relatively new, evidence for esophageal use is scarce and only few studies published on this topic, mostly for gastric cancer [21–26]. A case–control study implemented ICG during robot-assisted gastrectomy in 40 patients and showed a significantly higher LNY in patients in which the ICG technique was applied [21]. Future studies should investigate whether ICG could add to the LNY in clinical practice during esophageal surgery.

Although the benefit of robotic assistance for the abdominal LNY is not yet convincing, the added value of a robotic system for mediastinal LNY during transhiatal esophagectomy is more explicit. This is mostly the result of the ability to dissect up to a higher level in the mediastinum with robotic instruments compared to laparoscopic tools. Several studies reported on robot-assisted transhiatal esophagectomy and stated that the procedure is safe and feasible [27–29]. However, high quality evidence is lacking to conclude on the added value of robotic assistance due to the retrospective nature of the studies and small study populations with a maximum of 40 patients. In addition, no studies exist comparing laparoscopic to robot-assisted transhiatal procedures.

Besides transhiatal procedures, transcervical esophagectomy appeals on a robot-assisted lower mediastinal lymphadenectomy trough abdominal access. Transcervical esophagectomy is a new approach in which an esophagectomy could be performed by cervical access and thereby avoiding thoracic access with all its consequences (i.e., single lung ventilation, thoracotomy). In general, this procedure is combined with a robot-assisted transhiatal procedure, mainly for lymphadenectomy of the lower and or middle mediastinum [30–33].

## Short-term outcomes

### Postoperative complications

The effect of the surgical approach on postoperative complications in the abdominal phase during esophagectomy has been previously reported [34,35]. Laparoscopy has resulted in a significant reduction of pulmonary complications compared to laparotomy. However, the effect of robotic assistance on short-term outcomes during the abdominal phase has not yet been clarified and only few studies have compared short-term outcomes between robotic assistance in the abdominal phase and laparoscopy [16–19,36,37]. Contrary, a recent review is published on gastric cancer, including multiple cohort studies comparing robot-assisted gastrectomy to laparoscopic gastrectomy stating that robotic assistance during gastrectomy is (oncologically) safe [38].

For esophageal surgery, 1 study by Kwon and colleagues looked specifically to the abdominal phase with the aim to assess the added value of robotic assistance compared to laparoscopy [17]. In that study, the thoracic phase was performed by robotic assistance in both groups. After propensity scored matching, Kwon et al. included 49 patients in both groups with comparable baseline characteristics. Postoperative overall complication rate, anastomotic leakage and respiratory complications were equal between both groups. In addition, abdomen related complications including chyloperitoneum also did not differ between both groups.

All other studies compared fully robotic esophagectomy to video-assisted esophagectomy which involved thoracoscopy and laparoscopy. Consequently, it is hard to distinguish which phase, or combination, caused the outcomes.

None of these studies, found any significant differences in postoperative complications. Only Yang et al., comparing 271 patients in each group, reported a significant higher rate of liver dysfunction after thoracoscopic–laparoscopic esophagectomy (1.5% vs 0%, *p* = 0.045) [19]. In addition to postoperative complications, duration of hospital stay was equal between both groups in all the studies. Tagkalos et al. showed that intensive care stay was decreased by 1 day after fully robot-assisted esophagectomy (1 vs. 2 days, *p* = 0.029) when compared with conventional laparoscopy. However, this outcome was equal in the other studies [36].

Concluding, with the current evidence, robotic assistance in the abdominal phase during esophagectomy is not associated with a decrease nor increase in postoperative complications. However, no data regarding gastric conduit necrosis or leakage at the side of the staple line are reported. These outcomes could be relevant since they are related to the creation of the gastric conduit.

### Operation time

It is likely that duration of surgery will initially increase during the implementation of robotic assistance in the abdominal phase because of its novelty. Two studies compared the duration of the abdominal phase with robotic assistance and laparoscopy.

Tagkalos et al. reported a duration of 151 min with robotic assistance and 125 min with laparoscopy (*p* = 0.001) [36]. It is not mentioned if the potential learning curve was already completed or if the robot procedures are the first cases with robotic assistance.

Contrary, Deng et al. did not show a significant difference in duration of surgery between both groups (95 vs. 88 min, *p* = 0.096), whereas this study stated that the initial experience of robotic-assisted esophagectomy was reported [16].

Even though these data are ambiguous, it is conceivable that a slightly prolonged operation time could be expected when a new technique is implemented. Zhang et al. published the initial results of fully robotic McKeown esophagectomy in 72 patients and compared the results of the first 26 patients (group 1) to the following 46 patients (group 2) [40]. It took a median of 18 min (range 9–35) in the first 26 patients and 15 min (range 10–21) in the consecutive 46 patients for setting up and docking the robot in the abdominal phase (*p* = 0.015). The median abdominal console time was 67 min (range 40–235) in group 1 and 55 min (range 35–104) in group 2 (*p* = 0.003). In addition, specific CUSUM analyses for the learning curve regarding the duration of the abdominal phase were performed. The CUSUM plots revealed decreasing docking time at case 16 and decreasing console time after case 14.

### Proctoring

A dedicated proctoring program is essential for implementing a robotic system in esophageal surgery. The question whether it is safer to start with the implementation of a robotic system in the thoracic or abdominal phase is under debate. Based on the aforementioned studies reporting on short-term outcomes, it seems that adding robotic assistance to the abdominal phase effects duration of surgery during its implementation period but does not increase postoperative complications. In addition, CUSUM plots regarding operation time decrease at case 24 in the thoracic phase and case 14 in the abdominal phase [39]. The implementation of robotic surgery might be less risky in the abdominal phase compared to the thoracic phase since the abdominal phase involves less danger zones with vital structures. However, the abdominal phase also consists several difficulties. In particular, the creation of adequate exposure is challenging due to the multiquadrant surgical field. In addition, despite the new generation of robotic systems, colliding of the robotic arms is more common in the abdominal phase as compared to the thorax.

In favor of the thoracic phase, the surgery is more standardized and consists of a well-arranged step-by-step procedure which makes it suitable for a structured training pathway. Other factors playing a role in this debate are type of surgeon and their previous experiences with minimally invasive procedures. For instance, a general surgeon is likely to be more comfortable in the abdomen than a thoracic surgeon. Regarding experiences, the transition from conventional minimally invasive surgery to robotic surgery is generally smoother as compared to the transition of open surgery to robotic surgery.

In conclusion, when implementing a robotic system, the preferred phase to start proctoring differs per situation and experience.

### Jejunostomy

Jejunostomy placement is an optional step of an esophagectomy procedure, which is frequently performed to enable postoperative enteral tube feeding. Our recent literature review showed that jejunostomy tube feeding is probably preferable over nasoenteric tube feeding, as the former seems to be associated with less tube dislocations and better short-term quality of life [40–42]. However, jejunostomy-related morbidity should be acknowledged and prevented as much as possible. Intestinal torsion at the jejunostomy site represents a particularly troublesome complication, which has a reported incidence between 0 and 17% and usually requires a re-operation [43,44]. An attempt to minimize this complication is made by fixating the jejunum to the abdominal wall at a second fixation point with antirotation stitches.

When creating the jejunostomy, the camera should be repositioned to focus on the left side of the abdominal wall using the same port positioning. No redocking of the robotic arms is necessary. Only the camera position is changed from arm 3 to arm number 2 and the 30-degree scope is turned upwards. Arm number 1 is still used for the Cadiere forceps and arm 3 for the Needle driver (port of arm 3 is retracted to the abdominal wall to allow wristed movement to the near target). Arm 4 is not used in this phase. The use of a robotic system for creating a jejunostomy facilitates reliable purse string suturing of the jejunum to the abdominal wall using a barbed suture (V-Lock, Medtronic, USA).

**Fig. 4:**
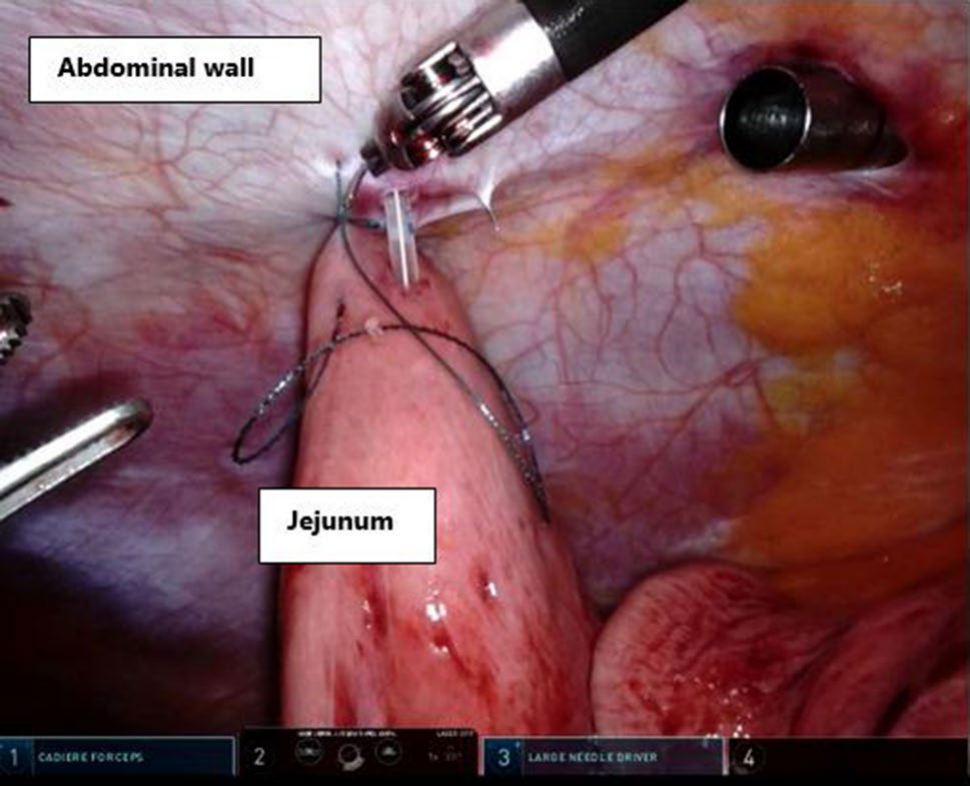
Robot-assisted creation of jejunostomy during esophagectomy using articulating instruments to fixate the jejunum to the abdominal wall. Robotic arm 1 is used for the Cadiere forceps, arm 2 for the camera, and arm 3 for the Needle driver. Arm 4 is not used

This jejunostomy technique can be challenging during conventional laparoscopy, since the working angle that is oriented towards the abdominal wall is not ideal. Robotic surgery increases the surgeons’ dexterity by the use of wristed instruments and thereby facilitates suturing during the jejunostomy placement (Fig. 4).

## Costs

The costs of a robotic system have been widely discussed. As discussed previously in this paper, it is unlikely that a robotic system will be specifically purchased for the abdominal phase during esophageal surgery. In case the robot is already used for the thoracic phase, it is probably cost saving to perform both the abdominal phase and the thoracic phase with robotic assistance. The same instruments could be used in both phases, whereas for laparoscopy an extra set of instruments is necessary. Besides surgical tools, it might be possible to reduce costs by saving on extra personal during the procedure.

However, the purchase of a robotic system and its maintenance remains expensive. For the thoracic phase, it might be suggested that these costs will possibly be equalized by reducing postoperative complications after robot-assisted esophagectomy compared to open esophagectomy [6]. With the current evidence, it is not likely that adding a robotic system in the abdominal phase will decrease postoperative complications as compared to laparoscopy.

Therefore, in terms of costs, robotic assistance in the abdominal phase is probably only cost saving if both thoracic and abdominal phases are performed with robotic assistance.

## Concluding remarks

The current evidence of the added value of robotic assistance in the abdominal phase is still minimal. The benefits rely mostly on surgeons’ perspective, for example, the independency of the surgeon and improved preciseness of dissection regarding stapling and lymph node dissection. The implementation of robotic assistance in the abdominal phase seems to be possible with a relatively limited learning curve and without compromising on short-term outcomes. Therefore, with the current evidence, robotic assistance in the abdominal phase during esophagectomy is at least not inferior to laparoscopy. Future prospective studies should reveal whether a robotic system will be worthwhile for the abdominal phase or even become superior over laparoscopy.
